# Trust and Uncertainty in the Implementation of a Pilot Remote Blood Pressure Monitoring Program in Primary Care: Qualitative Study of Patient and Health Care Professional Views

**DOI:** 10.2196/36072

**Published:** 2023-01-05

**Authors:** Evelyn Chew, Sok Huang Teo, Wern Ee Tang, David Wei Liang Ng, Gerald Choon Huat Koh, Valerie Hui Ying Teo

**Affiliations:** 1 National Healthcare Group Polyclinics Singapore Singapore; 2 Ministry of Health Office for Healthcare Transformation Singapore Singapore; 3 Saw Swee Hock School of Public Health National University of Singapore Singapore Singapore

**Keywords:** telemedicine, hypertension, remote blood pressure monitoring, health IT, primary health care, trust, health care provider relationship, blood pressure, primary care, qualitative study, health care workers, patients

## Abstract

**Background:**

Trust is of fundamental importance to the adoption of technologies in health care. The increasing use of telemedicine worldwide makes it important to consider user views and experiences. In particular, we ask how the mediation of a technological platform alters the trust relationship between patient and health care provider.

**Objective:**

To date, few qualitative studies have focused on trust in the use of remote health care technologies. This study examined the perspectives of patients and clinical staff who participated in a remote blood pressure monitoring program, focusing on their experiences of trust and uncertainty in the use of technology and how this telehealth intervention may have affected the patient-provider relationship.

**Methods:**

A secondary qualitative analysis using inductive thematic analysis was conducted on interview data from 13 patients and 8 staff members who participated in a remote blood pressure monitoring program to elicit themes related to trust.

**Results:**

In total, 4 themes were elicited that showed increased trust (patients felt reassured, patients trusted the telehealth program, staff felt that the data were trustworthy, and a better patient-provider partnership based on the mutually trusted data), and 4 themes were elicited that reflected decreased trust (patients’ distrust of technology, clinicians’ concerns about the limitations of technologically mediated interactions, experiences of uncertainty, and institutional risk).

**Conclusions:**

Managing trust relationships plays an important role in the successful implementation of telemedicine. Ensuring that trust building is incorporated in the design of telehealth interventions can contribute to improved effectiveness and quality of care.

## Introduction

### Background

An aging population worldwide and the consequent increased workload for health care systems have led to growing interest in technological innovations that can potentially lessen the strain on overtaxed health care systems [1,2]. Remote monitoring of blood pressure (BP) in patients with hypertension is an area that shows promise [3,4]. Studies to date featuring remote BP monitoring have reported positive outcomes in terms of acceptability and improved ability to manage one’s health, including in a predominantly minority, lower-income older adult population [5-7]. Home BP monitoring is now recommended as part of treatment in the clinical guidelines of several countries [8].

However, despite interest and enthusiasm on the part of health care providers, the mainstreaming and long-term sustainable implementation of such telehealth services is often fraught with challenges [9-13]. This is at least in part because the successful adoption and implementation of any telemedicine intervention depends heavily on human factors such as trust and acceptance, the lack of which can impede or even derail a program [9,10].

Trust is crucial to the health care provider-patient relationship and is very much at stake in the implementation of digital health. To begin with, the patient-provider relationship is fundamentally based on both interpersonal and institutional trust [14-16]. A trusting relationship with one’s health care provider is linked to better adherence to treatment and perceived effectiveness of care, whereas lack of trust is associated with lower rates of care seeking and appropriate treatment [15,17]. Telemedicine “necessarily alters the context of the traditional face-to-face physician-patient trust-based relationship,” in a shift that “may transform the substance of that relationship” [15]. Social shaping of technology theories tell us that technology design shapes user behavior; users, in turn, both shape and are shaped by the technology as they interact with it and within the larger system [18,19]. With regard to telemedicine, many questions arise: how does the patient-provider relationship change on an individual level because of the mediation of technology? How might the patient’s trust in the health care institution be affected? How much do patients and health care professionals trust the technology itself?

Presti et al [20] define trust as “an evolving, contextual and composite belief that one principal (trustor) has that another principal (trustee) will perform certain actions with certain expected results, when not all information about those actions is available.” More specific to e-services, the definition by Grandison [21] narrows this down to “the quantified belief by a trustor with respect to the competence, honesty, security and dependability of a trustee within a specified context.” Nevertheless, trust is a difficult notion to conceptualize and operationalize, and a vast array of conceptual categorizations and models of trust appears in the literature on trust and digital health, spanning psychology, management studies, IT studies, and health care research [21-30].

The early interdisciplinary model of trust by McKnight and Chervany [31] distinguished between dispositional, institutional, and interpersonal trust. Dispositional trust is intrapersonal, something that lies within a person; institutional trust is impersonal, grounded in situations or structure; whereas interpersonal trust refers to “trust in specific others.” In this early model, trust in technology is grouped under institutional trust.

The question of trust quickly rises to the fore in any technology-mediated service provider relationship. The technology acceptance model [32] initially focused on perceived usefulness and perceived ease of use but was soon expanded by researchers to include personal dispositions to trust, institution-based trust, and previous internet experiences, as well as user beliefs, attitudes, and intentions regarding the web-based environment [24,33,34]. Similarly, the Unified Theory of Acceptance and Use of Technology by Venkatesh et al [35] quickly expanded to include dimensions of trust. Elaborating on this model, Pal et al [36] found that, in the context of health care, perceived trust, technology anxiety, and expert advice were important factors for older adults’ acceptance of the Internet of Things and smart home technology. Deng et al [37], testing an extended version of the technology acceptance model, incorporated the role of trust and found that trust was the most important factor in patients’ adoption intention, whereas Arfi et al [29] found that perceived risk mediated perceived trust.

Finally, the eHealth Trust Model by Shen et al [25], which directly focuses on eHealth, integrates the Antecedent, Privacy Concern, and Outcome model and the Web-Trust Model [38]. In total, 6 antecedents to trust are listed: privacy experience, eHealth awareness, health care perception, demographic, technological savviness, and culture [25]. Clearly, trust is an important component of any system in which health, humans, and technology interact.

### Objectives

The intervention under study involved patients with chronic hypertension using a remote BP monitoring system to measure and upload their BP readings to a secure remote server, monitored periodically by the health care team. Follow-ups occurred through telephone consultations. This study sought to shed light on some ways in which such a program might positively or negatively affect trust relationships in health care.

**Figure 1:**
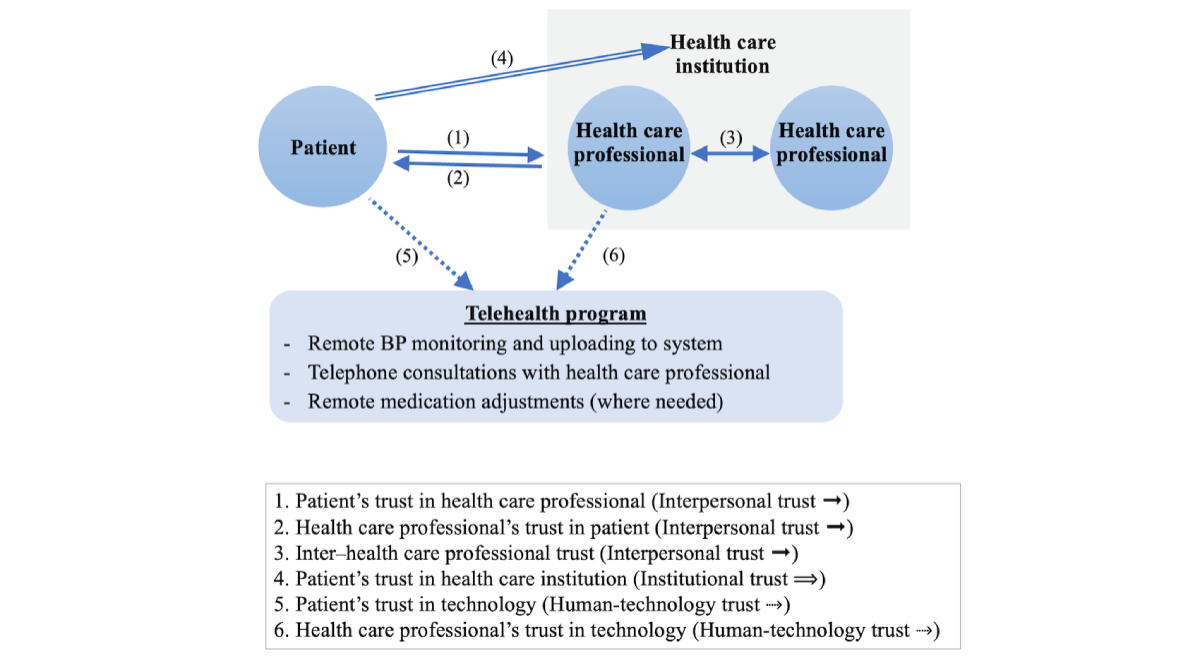
Trust relationships involved in the remote blood pressure (BP) monitoring telemedicine intervention.

Drawing from existing literature and observing the human and nonhuman actors involved in the telehealth program led us to deduce that 3 kinds of trust were of relevance: interpersonal trust, institutional trust, and human-technology trust [16,39,40]. Interpersonal trust refers to the trust between a patient and the individual health care professional. This trust is not only one-way, from patient to health care professional; in a home-based telehealth intervention, the professional must also trust that the patient will play their part. Moreover, interpersonal trust between health care professionals is involved when health care professionals must work together as a team in implementing the telehealth intervention. Institutional trust is the trust that patients place in the health care institution as a whole [41,42]. Human-technology trust [30] relates to patients’ and staff’s individual attitudes of trust toward the telehealth technology. The trust relationships implicated among the actors in this intervention are illustrated in Figure 1.

To date, few qualitative field studies have focused on the issue of trust in telehealth. Most existing studies on trust have carried out general surveys or built frameworks based on conceptual analyses. Thus, the value of this study was to use data available from our field quasi-experiment to extend existing findings about how trust in the patient-health care provider relationship is affected when a telehealth intervention is introduced.

Epistemologically, this study took a broadly critical realist and social interactionist approach [43,44]. Critical realism links the examination of structure and agency (germane to critical theory) with observable realities, thus remaining close to ground-level data. It also acknowledges that reality is an open system made complex by multiple, potentially nonreplicable causal mechanisms [45]. Social interactionism highlights that social realities are created and given meaning through human beings’ interactions with one another. Studying the question of trust in the patient-provider telehealth relationship through these lenses allowed us to interrogate social meanings and interactions and thereby elucidate the implications of such a program on trust in patient-provider relationships.

## Methods

### Overview

An interventional, quasi-experimental remote BP monitoring program was conducted in a polyclinic in Singapore from September 2018 to September 2019 involving 217 patients. Patients with hypertension were assigned to either a control arm or an intervention arm.

Patients in the intervention group were given a Bluetooth-enabled home BP monitor (TaiDoc Technology FORA P20b Blood Pressure Monitoring System) and a mobile data network–connecting gateway device (Phicomm Clue C230) that connected to a secure remote server. During a one-on-one in-person training session, patients were instructed on how to use the cuff and equipment to properly measure and upload their BP readings to the server. They were tasked to do this at least once a week from home over a period of 6 months to a year.

For the duration of the study, care managers who were nurses periodically reviewed the patients’ BP readings. Instead of in-person visits, patients whose BP was well controlled reviewed their condition through telephone consultations with their care managers (*scheduled teleconsultations*). If unexpectedly high readings were detected, care managers would contact patients to check on their well-being (*unscheduled teleconsultations*). Where clinically indicated, medications were adjusted over the phone after consultation with a physician. Quantitative and qualitative data were gathered and have been reported elsewhere (Teo, S, unpublished data, October 2022) [46]. This study is a secondary analysis of the qualitative interview data from patients in the intervention group.

### Setting

Singapore is a small, highly urbanized country in Asia with >5.7 million inhabitants. The country has an internet penetration rate of >81% [47], making it ideal for telemedicine, which is becoming increasingly popular [48]. The primary health care scene in Singapore comprises public and private institutions. Public health care is subsidized, with physicians in polyclinics—which are the public primary health care institutions—taking on a large share of the treatment of chronic illnesses [49]. Our study was set in a polyclinic in central Singapore.

### Participants

Participants were patients with hypertension from the intervention arm of the study [46] and staff who were involved in the program. Patient interviewees were referred by attending clinicians; staff involved in the research study were invited to participate by members of the research team. Patient participants had been in the remote BP monitoring program for at least 6 months. Of the 20 patients and 10 staff approached, 13 (65%) patients (n=8, 62% male and n=5, 38% female, with ages ranging from 35 to 73 years) and 8 (80%) staff members (n=2, 25% physicians; n=3, 38% care managers; n=1, 12% senior nurse clinicians; and n=2, 25% care coordinators) agreed to be interviewed. Participant demographics are listed in Tables 1 and 2.

**Table 1:** Patient participant demographics.

Participant ID	Sex	Age (years)	Education level	Occupation
F007	Male	47	Tertiary	Nursing home manager
F009	Female	47	Secondary	Cashier
F029	Male	50	Tertiary	IT manager
F021	Male	58	Tertiary	Teacher
F026	Male	49	Tertiary	Engineer
F035	Male	67	Secondary	Part-time consultant
F022	Male	64	Tertiary	Senior management
F096	Female	46	Secondary	Data entry clerk
F099	Female	64	Preuniversity	Not working
F118	Female	67	Primary	Part-time office cleaner
F119	Male	58	Preuniversity	Warehouse manager
F110	Male	35	Tertiary	Teacher
F122	Female	73	Tertiary	Not working

**Table 2:** Staff participant demographics.

Participant ID	Sex	Job title	Role in telemedicine program
S001	Female	Care coordinator	Train participants to use remote BP^a^ monitor; provide follow-up technical support
S002	Female	Care manager	Teleconsultation; monitor BP readings
S003	Female	Care manager	Teleconsultation; monitor BP readings
S004	Female	Family physician	Approve medication adjustments; counsel patient on medication changes
S005	Female	Care manager	Teleconsultation; monitor BP readings
S006	Male	Family physician	Approve medication adjustments; counsel patient on medication changes
S007	Female	Care coordinator	Train participants to use remote BP monitor; provide follow-up technical support
S008	Female	Senior nurse clinician	Support back-end coordination and implementation

^a^BP: blood pressure.

### Procedure

Interviews were carried out by study team members (female research fellows ECAL and TSH, who were experienced in qualitative research) face-to-face in a quiet room in the polyclinic before or after patients’ appointments. Patient participants were asked about their experiences of living with high BP; their thoughts on the telehealth program; and their experiences with the remote BP monitoring equipment, teleconsultations, and remote medication review (see Multimedia Appendix 1 for the interview topic guide). Staff participants were asked about their experiences with the telehealth program, focusing on their specific role (onboarding, teleconsultation, and medication adjustment) in carrying out the program. Patient and staff interviews lasted slightly less than an hour each. The interviews were audio recorded and transcribed verbatim. Interviewers had no direct or personal working relationship with interviewees apart from this study.

### Data Analysis

Using a Microsoft Excel (Microsoft Corp) spreadsheet and the transcribed interviews, an initial round of inductive thematic analysis was conducted by ECAL and TSH to elicit main themes from the interview data (Teo, S, unpublished data, October 2022). In the process, the question of trust arose as a theme that merited more detailed study. A secondary thematic analysis was run on the transcripts, focusing on the question of trust to further draw out other aspects of the theme. Secondary analysis of qualitative data is appropriate for cases where a researcher wishes to broaden and deepen in knowledge using data that have already been gathered [50-53]. As this is a secondary analysis, the principle of data sufficiency rather than data saturation was applied [54,55].

### Ethics Approval

The study was approved by the relevant institutional ethics board (Domain-Specific Review Board 2018/00785).

## Results

### Overview

We found 4 themes reflecting increased trust among the parties involved in the telehealth program and 4 themes that reflected reduced trust. Themes reflecting increased trust were as follows: patients felt reassured, patients trusted technology and the telehealth program, clinicians trusted the data generated, and a sense of partnership arose from the mutually trusted data. Themes that displayed reduced trust were as follows: patients’ distrust of technology, clinicians’ concerns about the limitations of technology, experiences of uncertainty, and institutional risk. Although it is not possible to list quotations from all participants, Textbox 1 lays out the themes along with the participants whose views support them.

**Themes elicited.**

- Themes reflecting increased trust
  Patients feel reassured that “someone is monitoring”: participants F007, F009, F035, F096, F110, F122, S002, S003, S005, and S006 (clinicians’ perceptions that patients felt reassured)
  Patients’ trust in technology and telehealth: participants F007, F021, F022, F029, and F035
  Clinicians’ trust in technologically generated data: participants S004 and S006
  Mutually trusted data support patient-clinician partnership: participants S004, S005, S006, S008, F007, F009, F026, F110, and F122

- Themes reflecting decreased trust
  Patients’ distrust of or discomfort with technology: participants F021, F099, F118, F119, S002, S006, S007, and S008
  Clinicians’ concerns regarding the limitations of technology-mediated interactions: participants S004, S005, and S008
  Experiences of uncertainty: participants F007, F009, F022, F029, F119, F122, S002, S005, and S008
  Institutional risk: participants S007 and S008

### Patients Feeling Reassured That “Someone Is Monitoring” (Interpersonal and Institutional Trust)

A dominant theme that emerged was that patients felt reassured that they were being closely followed up with by their health care team. This gave them a sense of security and increased their trust in both the health care professionals and the health care provider as an institution. Patients felt that they could “relax,” “knowing that at the other end, there is somebody looking at your [BP] readings” (participant F122):

I know that the [public healthcare institution] has my records and maybe if there’s any irregular kind of BP, hikes or something like that, they all might call me.

participant F007

I feel great, ya. At least I know that the polyclinic is keeping track of my blood pressure and then they’ll always make an effort to call us.

participant F110

A participant reported feeling “happy” when he was called by the clinic after submitting an unexpectedly high BP reading. He recognized that he was not alone in being concerned about his BP and felt supported in managing it:

...my blood pressure went suddenly went [up to] 150! They called me up...[laughs] they call me up, I feel happy!...If I do like [before the program], take [my BP reading as] 150, and just leave it behind, don’t care, 150! Then [if it were to] drag longer, then the blood pressure keeps on going up, [if there’s a] problem we don’t know also!

participant F035

Participants felt that they received more tailored guidance because of the intervention. Participant F035 added that, if not for the program, he would likely have ignored the high BP reading, lacking information on how to proceed, whereas the call from the clinic both reassured him and gave him specific steps to follow.

Another participant received a call the day after she submitted a high BP reading. She did not pick up the call as she was busy, leading the clinician to call several times. She was impressed by the swiftness and effort the staff made to contact her about her abnormally high BP, and this prompted her to take her condition more seriously:

When they called, then I know it’s actually serious for them.

participant F096

Others reported that the individualized guidance during the program on how and when to measure their BP correctly and avoid false measurements improved their ability to self-manage their condition.

The staff interviewees echoed this view:

The patients, most of them actually seem quite appreciative of it. Like they think it helps them—someone is monitoring, maybe it gives them reassurance. That we’re looking into readings.

participant S003, care manager

Compared to usual care, because [in] usual care you don’t really keep monitoring their blood pressure...whereas this [program], as and when you see slightly borderline high, you will just call. Ah, so they know that you are there.

participant S005, care manager

Patients felt supported in their health care management because of the perceived closer follow-up on the health care provider’s part. Interestingly, this trust was not linked to specific health care professionals but was often identified with the polyclinic staff as a whole using the generic pronoun “you all.”

### Patients’ Trust in Technology and Telehealth (Human-Technology Trust)

Among interviewees, attitudes toward technology—and, hence, trust in the remote BP monitoring program—varied widely. Some were enthusiastic about the usefulness of the program and appreciated the feedback from the BP monitor and the calls from the clinic, which helped them be more consistently aware of their health condition and its management; others were more apprehensive. The interviewees’ occupations also influenced what they thought of telemedicine. In total, 10% (2/21) of the interviewees—a manager of a nursing home and an IT professional—were particularly supportive of telemedicine and took a systemic view, arguing that technology not only could but ought to be leveraged to create efficiencies for the health care system:

A lot of things can be done by yourself, rather than needing a face-to-face with doctors. Sometimes you need [it], but not all the time. Sometimes online is good enough...If let’s say I have a particular question, if I can text or whatever some questions, [and] somebody can reply, [that] can be good as well.

participant F022

### Clinicians’ Trust in Technologically Generated Data (Human-Technology Trust)

In total, 100% (2/2) of the physicians interviewed appeared to trust the readings from the remote BP monitor more than the data generated by the previous system in which patients manually recorded their BP on paper. One physician noted that the remote BP monitoring data helped root out false “white coat” hypertension readings as patients’ readings taken at home would better reflect their BP in ordinary life. The other felt that the remotely generated readings were “more accurate” because of the following:

...they can’t alter it. The old [system], you can write down, you can erase it. You can write down a good reading, you can hide the high reading. So when they’re using the manual [record system], sometimes if I ask them further, they actually do have very high readings—they just don’t write it down. [Whereas the remote BP monitoring program takes] multiple measurements. So with this, in a way it’s more accurately reflecting their actual level of control for their blood pressure and...they can’t cheat.

participant S006, physician

### Mutually Trusted Data Support Patient-Clinician Partnership (Interpersonal Trust)

As the program required patients to provide sustained readings over a longer period, and as they were themselves involved in measuring and uploading the BP readings, patients themselves tended to trust the readings more than those recorded during their previous clinic visits. This enabled health care professionals to point to an objective and more accurate reading of BP over time, taken in situ in the environmental context of the person’s life.

A physician found that patients in this program were “more receptive” to advice and attributed it to patients’ “extra sense of security” in the patient-health care provider relationship owing to their active participation in the program, which resulted in increased interactions with the health care provider over 6 months:

Throughout the process of the six months of monitoring, it’s like a two-way thing. They submit the reading, high or low, we help to interpret. And it is not all the time that we push them to increase the medicine; sometimes...we give some compliments, throughout the process...it kind of strengthens the rapport, so they have higher level of trust, I guess...I just feel like it’s easier to talk to them about their [health] management when they come for the subsequent follow-up review.

participant S006, physician

As patients considered the BP readings they had submitted to be reliable and trustworthy, it was easier for physicians to present the data as evidence to persuade them to alter or begin a medication regimen when necessary:

...there are some patients whose [blood] pressure is always a little bit higher, but they always give excuses right? That it's their stress, they just came [to the clinic] and they were walking...and things like that. So when they go home and realize the [blood] pressure is also high at home, then it's a little bit easier to convince them that your [blood] pressure is not well-controlled and [there’s a] need to increase the medicine.

participant S004, physician

Some patients expressed stalwart support for the program, linked to implicit trust in the health care provider and awareness of their own role in actively managing their health:

I will never stop [participating in this program], because you know why? Right now the clinic is observing your blood pressure, anything [they will] call us, anything. Any problem, we will, you know, get the problem solved by the doctor overseeing.

participant F035

Actually [when I] signed on to this [program] I was thinking that uh—it’s a way that the polyclinic helps us to monitor blood pressure...in case that one day it really happens that we don’t know that we actually have blood pressure all the way [dangerously high blood pressure]. Because I think a lot of people, they aren’t aware that they do have [high] blood pressure. So this one can keep monitoring, so at least we got the awareness...I was thinking that it was quite a good project. So, have to try to take it up.

participant F009

In short, the remote BP monitoring program seems to have fostered greater partnership between the health care provider and patient via trust in the telehealth technology. As patients trusted the BP data that they had measured and uploaded, they also tended to trust the clinicians’ advice when those data were used as evidence to persuade them to engage in health-sustaining behavior.

However, for trust to grow, the initial rapport needed to have been established previously with a face-to-face consultation:

If I have a patient who has just transferred from a [private] GP and I put him on a machine, he won’t feel comfortable at all, I don’t think the patient will want to do that.

participant S006, physician

Lack of a previously established trust relationship resulted in longer teleconsultation time as the physician had to spend more time convincing the patient to follow their advice:

[When] rapport is not completely built up...it’s not as simple, even when you do the teleconsult titration [medication adjustment], over the phone we have to talk longer.

participant S006, physician

### Patients’ Distrust of or Discomfort With Technology (Human-Technology Trust)

The success of the aforementioned patient-provider partnership depends in large measure on human trust in technology—patients’ and health care professionals’ trust in the technological system in use. Where this trust is lacking, uncertainty and discomfort result. In total, 4 themes that negatively affected trust relationships (among patients, the health care provider, and the telehealth program) were patients’ discomfort with and distrust of technology, clinicians’ concerns about the limitations of the technology, uncertainties arising from lack of feedback from the program, and concerns about institutional risk.

Not all patients took to the BP monitoring device with enthusiasm; at least 15% (2/13) demonstrated ambivalence toward the program. Despite having agreed to participate in the telemedicine program, a few older patients became very nervous while interacting with the devices. Those who were hesitant about telemedicine found that experiences of failure or perceived failure to accomplish the task of uploading the BP readings correctly exacerbated their uncertainty and apprehension toward the telehealth program. For instance, a female participant aged 67 years was not used to technological devices and had to call the clinic when she forgot how to operate the device. Subsequently, she felt anxious and stressed each time she had to measure her BP, especially when she failed to distinguish between the different melody signals emanating from the BP monitoring system. She eventually dropped out of the program:

I started to give myself pressure. When it was time to measure my BP, I would become very nervous, I felt stressed. So my daughter said it’s better to drop out.

participant F118

For a minority (2/13, 15%) of interviewees, such as participant F118, who had only basic primary education, apprehension regarding the health care system and cultural beliefs and anxieties about seeing the physician were reflected in their reactions to the telehealth program. The same feature valued by some patients—follow-up calls from care providers—caused anxiety for these participants. Participant F099 would also become anxious whenever she recorded a higher BP reading or received a teleconsultation call. She associated calls from clinics and hospitals with bad news and would rather not hear from the health care provider at all:

If somebody call me, means something [is] wrong, I don’t like...So, if they don’t call me, it’s because my reading is good. If my reading is not good, they will call, definitely [to] ask me to increase my medicine.

participant F099

Moreover, at least one patient (participant F021) felt a need to present a positive result to the health care provider as he felt that the initial higher reading that he obtained was not reflective of his typical BP and he did not want the clinic to call him. To ensure that a good reading was uploaded, he first would measure his BP using his own BP monitor and then repeat the process with the remote BP monitor provided by the polyclinic only when the readings were favorable.

Some patients embraced certain aspects of the program but not others. Although they valued the “extra sense of security” of having their BP monitored remotely (5/13, 38%), some patients (2/13, 15%) disliked the aspect of remote phone consultations replacing physical visits. They lacked trust in the validity of a remote telephone consultation and felt safer seeing a physician face-to-face. A phone consultation was considered a dubious and poor substitute for an in-person consultation. As a result, clinicians reported that some patients would call in to cancel their teleconsultations and show up to the clinic instead for their routine consultations, as they used to do before the program. As one physician (participant S006) pointed out, this was contrary to the purpose of the program, which sought to reduce clinic visits via self-management and remote BP monitoring.

In a similar vein, some patients were reluctant to increase the dosage of their medications over the phone as they lacked confidence in the remote consultations. This was reported by 15% (2/13) of the patient interviewees and 12% (1/8) of the staff interviewees. A staff participant opined that this was because “they don’t see you” (participant S002, care manager). Reflecting a deep-seated uncertainty as to the trustworthiness of remote telephone consultations, an interviewee who rejected the possibility of having his medication adjusted over the phone explained the following:

...If let’s say, they want to increase [my dosage], I would rather come and meet and find out why I need to increase...We are not sure, doctors are busy also. Did they make a mistake or not? This—that is a phobia. Did they make a mistake? You know? Or it may be somebody’s information, but you called the wrong person. So I will—as far as medication is concerned, for my health or any disease I’m suffering, I’d rather have face-to-face...Sometimes, certain things, I don’t feel comfortable talking on the phone.

participant F119

Finally, a few (3/13, 23%) patients shared their cybersecurity concerns—where their data would be stored and the possibility of leaked personal information. A patient pointed out the possibility of impersonation over the phone, that a scammer or prank caller might pretend to be a health care professional:

Over time people might, you know, exploit this loophole. People try to imitate and then mess up your life. And then tell you, [that you’ve] got to take four pills instead of one.

participant F029

However, the same patient had professional experience in IT and himself suggested the solution of implementing 2-factor authentication or a confidential identifier code to verify the health care professional’s identity. Overall, concerns about cybersecurity surfaced infrequently in our interviews; most interviewees expressed trust in and a positive attitude toward the use of technology and telemedicine.

### Clinicians’ Concerns Regarding Limitations of Technology-Mediated Interactions (Human-Technology Trust)

Mirroring patients’ uncertainties about whether telemedicine could provide the same level of care as an in-person consultation were clinicians’ concerns about the teleconsultations. Clinicians’ apprehension centered on the inability to ascertain if their messages were correctly received by patients over the phone:

...you must really make sure that they understand...Sometimes [when] you talk, you think you are quite clear, but the other party’s hearing is a bit [impaired]. And then, they don’t know what you asked them to do.

participant S005, care manager

Participant S004, a physician, agreed that “sometimes it’s a bit dangerous to do things over the phone” and felt that teleconsultations should be reserved for patients with greater health literacy and adequate social support to avoid miscommunication. Particularly with medication adjustments, a clinician worried about the extended time between in-person visits:

We still want them to come back, we still want to see them [to find out] whether they’re taking [their medications] or not, if there are side effects, do they know when to stop...

participant S005, care manager

The potential for miscommunication over the phone was also greater than in face-to-face consultations:

We can’t see the body language. Face-to-face, if I know that you are not paying attention to what I say, then I have to repeat, repeat...But if over the phone, I cannot [be sure] that you are actually listening correctly...Then [they] may end up taking [the medication] wrongly.

participant S008, senior nurse clinician

A care manager noted that it was easier to build interpersonal rapport, elicit information about lifestyle and medication compliance, and clarify doubts with the patient in person. She also highlighted that some patient caregivers were worried about unclear communication over the call and, therefore, would prefer to avoid teleconsultations.

In short, some patients and some staff interviewees had concerns about the limitations of a teleconsultation compared with a face-to-face encounter. Moreover, despite acknowledging the advantages of time savings and convenience, a few interviewees among both staff and patients felt that in-person visits provided more information than telephone consultations.

### Experiences of Uncertainty (Human-Technology Trust)

Feelings of uncertainty regarding diverse aspects of the telehealth program marked several interviewees’ responses among both patients and staff. In total, 3 aspects were identified: lack of visibility (clinicians), lack of feedback from the telehealth system (patients), and lack of feedback from health care professionals (patients).

For health care professionals, uncertainty arose from the lack of visibility of certain information owing to the properties of the telemedicine technology. For instance, from the BP readings on the back end of the system, care managers were unable to ascertain the “why” of an abnormal reading—seeing only the BP measurements, they did not know if patients’ high readings were the result of exercise rather than disease. When a participant failed to upload their readings, they were unable to verify whether the readings had indeed been taken but were not transmitted owing to a technical glitch or whether the patient had neglected to do the requisite BP monitoring. Therefore, patients’ irregular submission of data caused concern for clinicians:

Some patients submit readings irregularly, then at the back end, I worry whether the patient is having any problems...Then I start to call them.

participant S002, care manager

For patients, lack of feedback from the system surfaced as a design flaw in the BP monitoring device that created uncertainty and discomfort. For instance, uncertainty over whether the readings had been uploaded to the server led some patients to send in several readings in a row, leading to multiple recorded readings that mystified the care manager in charge:

...I asked the patient out of curiosity, “How come you measured your blood pressure so many times in a minute? Or in five minutes so many readings?”...They say it seemed like the reading was not transmitted, so they kept re-measuring, re-measuring, re-measuring...so the numbers keep transmitting to us and we get a lot of readings...and I cannot stop them, because it might be true [and] if I stop this practice, I might get no reading here in the end.

participant S005, care manager

For one participant, uncertainty arose from the lack of a channel to clarify her medical doubts when side effects occurred after having her medication adjusted:

So last week I took the new pill which is a tablet, I kind of feel a bit strange, uh not—not...there’s something that I cannot explain...I would prefer that there is a contact that I can call. Because teleconsult—through telephone, they may just say, okay you just take and that’s it. But what if I take and I don’t feel quite right?...So my point is, if we are going to go through this program, we won’t come back until maybe six months later or some time, that could be a bit too far, especially if I’m on new medication.

participant F029

In short, both patients and staff experienced uncertainty. For health care staff, this was related to the limited information provided by the telehealth system and the incomplete picture they were able to form of the patient’s state of health. For patients, it was related to limited feedback (from the telehealth system and the uploading process) and to the inability to clarify doubts about their health condition.

### Institutional Risk (Institutional Trust)

The health care professionals we interviewed were acutely aware of the risk that a failure in the accuracy of the remote BP equipment might pose to patients’ trust in the health care institution as a whole. A few patients occasionally noted discrepant readings between their own BP monitoring devices and the study equipment, which caused some concern among staff. A staff interviewee was worried that “for anything that turns bad, there might be negative impact, like they lose trust in our treatment because the devices don’t work well or it’s not as accurate as it should—they expect it to be” (participant S007, care coordinator).

Clinicians were also somewhat concerned about overblown patient expectations of what the telehealth program could achieve. For instance, some patients might expect an immediate response from the medical team in the case of unexpectedly high BP readings, which could indicate a medical emergency. Failure to respond quickly in such a case might result in grave medical consequences as well as disappointment and distrust in the health care provider. To prevent such a situation from happening, staff reminded patients of the limits of the program. A staff interviewee stated emphatically that, if 2 subsequent readings were abnormally high, patients should “always give us a call immediately...like, do not wait, *do not wait* for our call because it is not real time monitoring. And we always emphasize, [it is] not real-time” (participant S007, care coordinator). Although these guidelines were primarily geared toward patient safety, health care staff were also aware of the reputational risk for the institution implied in the telehealth program.

## Discussion

### Principal Findings

Our analysis showed that patients and staff both felt that the telehealth intervention had an overall positive impact on interpersonal and institutional trust in the patient-health care system relationship. The telehealth intervention was generally well received—patients felt reassured and trusted the technology, clinicians trusted the technology and the patient-generated data, and this enabled greater partnership in patients’ health management. Nevertheless, the intervention also surfaced some underlying anxieties and concerns that patients and staff alike had about the telehealth intervention, viz., some patients’ distrust of or discomfort with technology, clinicians’ concerns regarding the limitations of technology-mediated interactions, patients’ and clinicians’ experiences of uncertainty, and institutional risk. Given the age distribution of patient interviewees as predominantly 40 to 70 years, it should be noted that the findings may reflect the views of this particular demographic, which may differ from the views of younger patients.

The most salient themes in the data related to patients’ trust in individual health care professionals, in the health care institution, and in technology (relationships 1, 4, and 5 in Figure 1) and to clinicians’ trust in technology (relationship 6 in Figure 1). Table 3 summarizes the findings.

**Table 3:** Summary of themes and trust relationships.

Relationship	Type of trust relationship	Trust-facilitating theme	Trust-hindering theme
Patient-health care professional (1 and 2)	Interpersonal trust	Patients feel reassured that “someone is monitoring”	—^a^
Patient-health care institution (4)	Institutional trust	Patients feel reassured that “someone is monitoring”	Institutional risk
Patient-telehealth technology (5)	Human-technology trust	Patients’ trust in technology and telehealth	Patients’ distrust of or discomfort with technology Patients’ experiences of uncertainty
Health care professional-telehealth technology (6)	Human-technology trust	Clinicians’ trust in technologically generated data	Clinicians’ concerns regarding the limitations of technology-mediated interactions Clinicians’ experiences of uncertainty
Patient-telehealth technology-health care professional (1 and 2)	Interpersonal trust Human-technology trust	Mutually trusted data support patient-clinician partnership	—

^a^No themes emerged from the data in this category.

### Telehealth as Supplementary Rather Than Substitutive

Although some researchers [15,56] have raised concerns that depersonalization could occur and trust relationships would be damaged with the adoption of telemedicine—the “transformation of the fiduciary relationship into a more contractual or quasi-contractual relationship” [15]—we found the reverse to be true. Increased contact with the health care system, albeit remotely via patients’ participation in uploading their BP readings and teleconsultations, seems to make the health care provider more continuously present to the patient than before. Patients felt that they were more closely followed up with and were more aware of health care providers as partners in managing their health. However, as a physician observed, the trust relationship needed to be properly established in person before telemedicine was introduced. This is consistent with observations by van Middelaar et al [57] and others [58] that an eHealth intervention is more readily trusted when eHealth is combined with at least an initial in-person interaction with a trusted offline entity at the outset.

Our study’s findings suggest that telemedicine is not necessarily detrimental to the human touch or bedside manner of the physician, as some critics fear. In fact, the generally positive feedback indicates that telehealth has much potential to supplement (though not totally replace) face-to-face health care by extending the care and attention given by health care providers beyond the physical boundaries of the clinic.

### Remote BP Monitoring Creates Ubiquitous and Continual Presence of the Health Care Provider

From a patient perspective, the extension of care beyond the walls of the polyclinic via the remote BP monitor and teleconsultations blurs the boundaries of care, blending the world of clinical treatment with the intimacy of patients’ daily lives. The elements that constituted the telehealth program—the physical presence of the telehealth equipment, phone calls from the health care provider, and patients’ action of uploading their readings weekly—engaged patients in their own care and transformed the health care provider-patient relationship from a predominantly episodic one bounded by time (of the patient’s appointment) and space (the venue of the polyclinic) to one that formed part of the fabric of their ordinary lives, leading to a greater sense of trust in the health care provider. Thus, patients’ experience of health care shifted from being periodic and transactional to an ongoing, continual relationship with the health care provider virtually present in their homes by means of remote BP monitoring and telephone consultations.

Over time, because of the technologically mediated interactions with the health care system and telephone consultations, patients felt greater familiarity with the health care professionals. As a result, some patients appear to have more readily accepted physicians’ advice on medication adjustment.

Trust transference from the in-person context to the technological one is likely to have played a key role in the first 2 themes as all patients enrolled in the study had previously engaged in face-to-face encounters with clinicians at this health care facility, although not necessarily with the same clinician. Several studies have highlighted the existence of trust transfer in eHealth, “from brick to click.” Van Velsen et al [59] found that, for patients, trust in the care organization was conceptually different from trust in the care team and trust in the treatment but that trust in the care team and trust in the treatment affected trust in the technology. Our findings also support those of Meng et al [60] and Pavlova Miller [40], who found that trust in offline health services was positively associated with trust in web-based health services.

### Selective Uploading and Naïve Trust in Systems

Interestingly, the health care professionals interviewed tended to perceive the technologically mediated readings as more reliable than manually recorded ones, on the assumption that patients “can’t cheat” because the BP reading is automatically uploaded to the system. However, some patients sought to control or manipulate the BP readings uploaded so that only desirable readings would be sent to the system. This implicit trust in technology observed from the health care professionals was at odds with the discovery that patients may in some cases modify what is recorded to selectively present their biodata. Choosing to upload only desirable readings may be attributable to a form of social desirability manifesting itself as the desire to present oneself as a “good patient” [61-63]. Therefore, it is pertinent for health care providers using telehealth to be aware of the role human factors and motivations play in patient behavior. They will in this way avoid assuming that data uploaded by patients and produced in the context of patients’ lives occurs in a completely objective environment devoid of subjective and extraneous influences.

### Managing Uncertainty and Risk

Our key themes reflected various aspects of both trust and uncertainty associated with telehealth technologies. Uncertainty is antithetical to trust; trust and uncertainty have been described as “a pair of opposing forces shaping relationships” [64] in dialectical tension. To decrease uncertainty is to help foster conditions necessary for trust.

Our study showed that uncertainty was often present in the telemedicine interactions. For patients, there was uncertainty about where the biodata would be stored, lack of feedback on whether the BP measurement uploading process was successfully completed, if and when the submitted data were being monitored by the health care provider, and the inability to clarify doubts about their medical conditions or medications.

The interviewees who distrusted technology became anxious about their ability to successfully upload their readings (ie, low technological self-efficacy) and tended to be more likely to drop out of the program. This is in line with previous findings that users’ postadoptive behaviors are affected by trust in the technology and that technological self-efficacy may be a mediating variable for trust in technology [65]. Greenhalgh [66] points out that technological innovations often fail because “the patient in the guideline does not correspond to the patient in the bed”—telemedicine initiatives often envision an empowered, self-motivated patient who understands, trusts, and happily uses the technology. Our study found this to be true of many patients, but others, especially certain patient interviewees aged >50 years, were distrustful of or apprehensive about technology. For this program to be effective and sustainable in the long run, additional efforts should be made to reduce uncertainty and raise the level of comfort with the telehealth technology for such segments of the population.

For clinicians, uncertainty arose from the lack of visibility of a patient’s actual health status because of the limited data circumscribed by the properties of the technology used. To increase the sustainability and acceptability of this program, it would be helpful to increase the trust of all human stakeholders by reducing the uncertainties faced by patients and staff. Design choices such as having clear feedback from the device when the reading has been correctly uploaded and explicit indications of how often the patients’ uploaded data will be reviewed could go some way toward reducing uncertainty for patients. Reducing uncertainty would make it easier for staff and patients to trust the technology and for patients to increase their confidence in the telehealth program as well as in the health care provider.

Managing institutional risk and the uncertainties it causes to organizational stakeholders is also important for any telehealth program. Support can be provided through clear guidelines, as was done in this case, to limit uncertainties about legal liabilities, reputational risk, and other repercussions on health care professionals arising from possible failures in the telehealth program.

### Limitations

This study undertook an exploration of user views during the implementation process, supplementing the findings from a larger mixed methods study. Slightly more than half (7/13, 54%) of our interviewees were aged >50 years, reflecting population prevalence as hypertension is much more common in this age group [67]. More work is needed to understand the needs of younger patients with early onset hypertension as they are likely to engage differently with telehealth.

As this study interviewed patients enrolled in a remote BP monitoring program, some selection bias is to be expected as potential participants who are deeply averse to technology would have declined to participate. Future studies should specifically seek out views of segments of the population who are less inclined toward telehealth to elucidate their concerns.

### Conclusions

In our study, telemedicine was used to complement existing face-to-face care by reducing physical clinic visits while increasing the monitoring of patients’ health via technology-enhanced remote BP monitoring. Our findings elicited aspects of patient trust in health care providers (as individuals and as an institution) as well as in the telehealth technology and found elements that encouraged or hindered the building of trust in an existing patient-health care provider relationship. Generally, patients greatly valued the closer follow-up, which was also deemed more personal, although a few refused to relinquish or reduce in-person follow-up visits. Future work could investigate the possibilities of telemedicine to *extend* the human touch in remote medical care rather than substitute it. Well-designed telehealth interventions can remotely extend the presence and sense of closeness of the health care provider and, thus, increase quality of care without detriment to productivity, resulting in stronger partnerships with patients in managing their health.

Other aspects of the trust relationships warrant further research, such as how the affordances and design of a specific telehealth intervention affect perception and trust, the impact of telehealth on interprofessional trust relationships, and how telehealth affects the health care provider-patient relationship in the absence of a previous offline relationship.

Attitudes toward new technologies are often mixed, with some stakeholders enthusiastic about the novelty and others critical, skeptical, reluctant, or even hostile [9,10]. However, for telemedicine to work well, trust is crucial [68]. Exploring the impact of a telehealth intervention on trust relationships helps shape future developments of similar projects with a view to maximize benefits, avoid pitfalls, and enhance patient relationships with their health care providers. It is hoped that this exploration of the dimensions of trust in a telehealth program will assist designers and implementers of telehealth as well as health care researchers in taking cognizance of the role of trust and other human factors in their telehealth program development and its implementation.

## Appendix

Multimedia Appendix 1 Patient and staff interview topic guide.

## Abbreviations

BP: blood pressure
