# Supplementary material for: Trust and Uncertainty in the Implementation of a Pilot Remote Blood Pressure Monitoring Program in Primary Care: Qualitative Study of Patient and Health Care Professional Views
Source: JMIR Hum Factors. 2023 Jan 5;10:e36072. doi: 10.2196/36072 (PMC9853336; doi:10.2196/36072)
Supplement: Multimedia Appendix 1 [file humanfactors_v10i1e36072_app1.pdf]

Multimedia Appendix 1: Patient and staff interview topic guide

INTERVIEW GUIDE (PATIENTS)

Opening Questions

- Can you tell me what it is like for you to have high blood pressure?
- Can you tell me why you signed up for this study?
- Can you tell me about your experience of your chronic disease management with your care team?

Experience of participating in the study

- Can you share about what you liked about the study?
- Can you tell me about anything you did not like?
- Were you monitoring your blood pressure before entering this study?
- Can you tell me about your experience of monitoring your blood pressure using telemonitoring equipment? How is this different from what you have been doing before entering this study?
- Can you share about your experience with using the gateway device?
    - Time taken to start the device
    - Usability
- Have you used the BP machine outside of home? Can you tell me your experience?
    - Have you used it overseas?
- What do you feel are some of the advantages of telemonitoring of blood pressure?
- What do you feel are some of the disadvantages?
- How do you feel about your blood pressure now?
- How do you feel about the control of your blood pressure now?
- Has participating in this study made you more aware of monitoring your blood pressure?
- Should this programme becomes available permanently, would you choose to continue using:
    - the telemonitoring equipment and self-monitor your blood pressure? Why or why not?
    - the teletreatment (consultation and medication adjustment) and self-monitor your blood pressure? Why or why not?
- Would you recommend this telehealth programme to other people with high blood pressure? Do you have any suggestions for improvement? Please elaborate.

INTERVIEW GUIDE (STAFF)

Experience of participating in the study

- I am interested in hearing about your experience of providing care to the participants of this study, can you tell me all about it?
  - Can you tell me about your role in this study?
- How is your experience different from usual care? Can you share what you liked about the study?
- Can you share what you did not like about the study?
- What would be some of the advantages of telehealth (BP monitoring, teleconsultation, medication adjustment) in the management of blood pressure?
- Can you share some of the disadvantages?
- How has your experience been with patients under this study?
  - Could you elaborate about how this study affected relationship between patient and the healthcare provider?
- Did you have any interesting experiences arising from the study?
- Do you think patients would continue using telehealth to manage their blood pressure if it was still available after this study? Why or why not?
- Would you recommend telehealth to manage blood pressure to your patients with high blood pressure?
- Do you have any suggestions for improvement? For e.g. when and under what situations would a chatbot be useful? Please elaborate.

Onboarding (For Care Coordinators)

- How was your experience in enrolling patients for this study?
- How did you find the online portal used in the study? Regarding navigation, speed, format, connectivity, etc.
- How was your experience in teaching patients about using the BP machine?
- What were some of the difficulties that patients faced?
- What were some of the difficulties that you faced?

Teleconsultation Experience (For Care Managers)

- How was your experience of teleconsultation with your patients?
- Can you share what you liked about teleconsultation sessions?
- Can you share what you did not like about teleconsultation sessions?
- What would be some of the advantages of teleconsultation?
- Can you share some of the disadvantages?

- How was interacting with patients via this platform as compared to usual clinic visits?

Medication Titration Experience (For Doctors and Care Managers)

- How was your experience of medication titration in this study?
- Can you share what you liked about medication titration in this study?
- Can you share what you did not like about medication titration in this study?
- What would be some of the advantages of this way of titrating medication of hypertensive patients?
- Can you share some of the disadvantages?
- Would you recommend your friends or family to enrol in a similar programme? Why or why not?
- What do you think is the value of this programme to patients?
- Is there anything else that you would like to add that is not already covered?
